# Predicting the Incidence of Human Cataract through Retinal Imaging Technology

**DOI:** 10.3390/ijerph121114800

**Published:** 2015-11-19

**Authors:** Chi-Ting Horng, Han-Ying Sun, Hsiang-Jui Liu, Jiann-Hwa Lue, Shang-Min Yeh

**Affiliations:** 1 Medical Education Center, Kaohsiung Armed Forced General Hospital, Kaohsiung City 802, Taiwan; E-Mail: h56041@gmail.com; 2 Department of Pharmacy, Tajen University, Pintung City 90741, Taiwan; 3 Institute of Biochemistry and Biotechnology, Chung Shan Medical University and Chung Shan Medical University Hospital, Taichung City 40201, Taiwan; 4 Department of Optometry, Chung Shan Medical University, Taichung 40201, Taiwan; E-Mail: hanying@csmu.edu.tw; 5 Department of Ophthalmology, Chung Shan Medical University Hospital, Taichung 40201, Taiwan; 6 Department of Optometry, Mackay Junior College of Medicine, Nursing, and Management, Taipei 112, Taiwan; E-Mails: s458@eip.mkc.edu.tw (H.-J.L.); t106@eip.mkc.edu.tw (J.-H.L.); 7 Department of Optometry, Central Taiwan University of Science and Technology, Taichung 40601, Taiwan

**Keywords:** cataracts, pathogenesis, retinal imaging technology, ray tracing, periodic hole array

## Abstract

With the progress of science, technology and medicine, the proportion of elderly people in society has gradually increased over the years. Thus, the medical care and health issues of this population have drawn increasing attention. In particular, among the common medical problems of the elderly, the occurrence of cataracts has been widely observed. In this study, we developed retinal imaging technology by establishing a human eye module with ray tracing. Periodic hole arrays with different degrees were constructed on the anterior surface of the lens to emulate the eyesight decline caused by cataracts. Then, we successfully predicted the incidence of cataracts among people with myopia ranging from −3.0 D to −9.0 D. Results show that periodic hole arrays cause severe eyesight decline when they are centralized in the visual center. However, the wide distribution of these arrays on the anterior surface of the lens would not significantly affect one’s eyesight.

## 1. Introduction

An eye that has no refractive error when viewing distant objects is said to have emmetropia or be emmetropic, which means that the eye is in a state in which it can focus parallel rays of light (light from distant objects) on the retina, without using any accommodation. A distant object in this case is defined as an object that is 8 m or further away from the eye. This has proven to be an evolutionary advantage as it automatically focuses the eye on objects in the distance, thus allowing an individual to be alert, for example, in a prey-predator situation. The lens of the eye focuses the light directed from the pupil onto the retina where nerves then carry the images to the brain. The lens can change shape depending on the distance of the object being focused on.

Cataracts is a kind of disease that causes visual disorders resulting from lens opacity. This medical condition can generally be divided into two types: congenital and acquired. Senile cataracts is the most common variety of cataracts and is an aging phenomenon. In this case, the lens of the eye slowly hardens and becomes opaque with age. Statistics indicate that the incidence rate of cataracts is 60% for people over 50 years, 80% for people aged 60 and over, and up to 90% for people aged 70 and older. Therefore, senile cataracts is a disease that commonly occurs among the elderly. In the United States, over 0.4 million patients annually undergo surgery because of cataracts [1,2,3,4]. In the early stages of cataract formation, blurred vision, tone change, photophobia, black spots, diplopia, and myopia resulting from cataracts may be observed. As a cataract advances, visual disorders increase and, eventually, patients can only distinguish fingers placed right in front of their eyes or can gaze at light using only one eye [5].

The various types of cataracts occur due to several reasons. The most common form of cataracts, senile cataracts, occurs with increasing age. In particular, these cataracts begin to occur in people in their 40s or 50s, when the lens slowly hardens and becomes opaque, thereby resulting in visual disorders among this group. Traumatic cataracts are caused by traffic accidents, stab wounds with sharp objects, or penetrating intraocular drugs. Complications such as iritis and glaucoma can lead to a cataract, while metabolic cataracts can arise because of diabetes and diseases of the thyroid gland. Drug-induced cataracts occur with the long-time administration of steroid drugs. In comparison, congenital cataract is caused by genetic diseases, chromosome variation, and intrauterine infection [6]. In this cataract, white or gray muddy spots can be observed in the pupil of infants with poor visual development [7,8,9,10].

In a previous study, we examined corneal surgery for vision correction via the technique of multi-layer swim-ring-shaped wave circles through optical simulations with the use of a Monte-Carlo ray tracing method known as Advanced System Analysis Program (ASAP). Simulation results showed that the ability of the crystalline lens to adjust increased tremendously from 1 D to 4 D. The method was also used to compare the images displayed on the retina before and after treatment. The results clearly indicated a significant improvement in presbyopia symptoms with the use of this technique [11]. As a way to resolve this medical problem, the current study aims to add a microstructure to the crystalline lens in the eye. The microstructure is designed to irradiate light into the eye, causing scattering. The periodic structures will then cause vision decline because of scattering. Based on the deterioration of eye vision, we can emulate a situation similar to a cataract disease through an ASAP optical computing model applied to the microstructure. Consequently, based on the ASAP Snellen chart, the flux distribution is emulated in different degrees of myopia. The fluxes featuring different degrees of myopia are matched to the computing result with the microstructure, in order to determine the degree of myopia that matches the most. The degree of myopia is then used to predict the severity of a patient’s lens disease.

## 2. Materials and Methods

**Figure 1:**
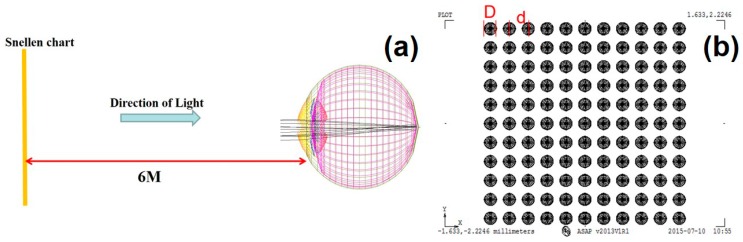
(**a**) Research framework chart; (**b**) schematic diagram of the microstructure placed on the anterior surface of lens.

Figure 1a shows the experimental framework of this research. A Snellen chart is placed 6 ft away from a human eye. Light is beamed onto the eyeball from the Snellen chart, which is imaged in the retina. The image can reappear in the retina through our optical computation. The structure of the human eyeball includes the cornea, iris, pupil, crystalline lens, and so forth. The cornea, a kind of transparent membrane with a diameter of about 11.5 mm and a thickness of 0.5 mm–0.6 mm in the center and 0.6 mm–0.8 mm at the periphery that covers the eyeball, is located in the most lateral part of the eye. The cornea refracts and collects the light at the fundus. 

**Figure 2:**
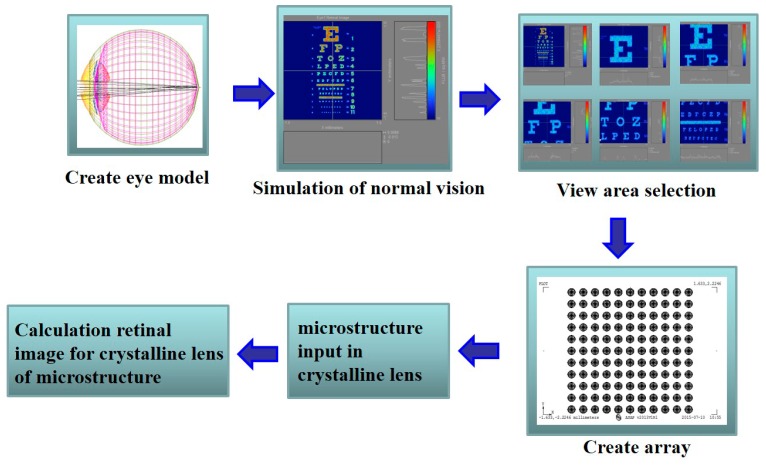
Research flow chart.

Apart from protecting the eyeball, the cornea is also the first line of defense for refracting the parallel rays of light entering the eyes. The iris exhibits a circle surrounding the pupil, a condition similar to the aperture of a camera. The iris can adjust the size of this circle according to the external light intensity. In other words, the pupil automatically shrinks in bright places and automatically magnifies in dark ones. The iris adjusts the entry of light into the eye. The pupil is a black region in the center of the iris. The crystalline lens is transparent with a diameter of approximately 1 cm and usually has the shape of a Chinese chess piece. The convex lens can become thin or thick to refract the light and focus it in the retina. A typical Snellen chart displays a row of letters representing a visual acuity of either 20/20 or 20/200. In this case, 20/20 represents 1.0, whereas 20/200 depicts 0.1. This measurement implies that human beings can distinguish an object sized 5 arc min (1 degree = 60 arc min) 20 feet outside or can identify the line in the interval of 1.75 mm. Hence, 20/20 is the standard human vision. If one’s vision is 20/200 (0.1), then he/she cannot clearly see the object 20 feet away from his/her eyesight compared with a person with the standard vision (20/20 or 1.0). In this study, some microstructures are added to the crystalline lens of the eye to emulate the situations similar to a cataract, in order to identify the most consistent degree of myopia through light intensity distribution in the ASAP Snellen-chart. Figure 1b shows a schematic diagram of the microstructure placed on the anterior surface of the lens. This microstructure is about 10 μm in size. In the figure, *D* is the diameter of each microstructure, and *d* is the interval between the center of one microstructure and another. The size and distribution density of the grain can be modified. For example, for a 50 × 50 or 100 × 100 grain, the number of the levels on the surface of lens can be set. Moreover, the arrangement instruction of random numbers in ASAP can be used to make the microstructure arrange the random numbers. Figure 2 shows our research flow chart. First, an eyeball module is established, after which a Snellen chart under normal vision emulated by ASAP is used to calculate its light intensity. Next, different regions in the Snellen chart are selected to emulate myopia conditions ranging from normal vision to −10.0 D with light intensity calculation, respectively. The data are presented as a trend line. Then, the microstructure is established, and the required parameters are set. The established microstructure is placed on the lens. Finally, we emulate the changes in the vision after placing the microstructure to compare the results with original vision to determine the corresponding power.

## 3. Results and Discussion

**Figure 3:**
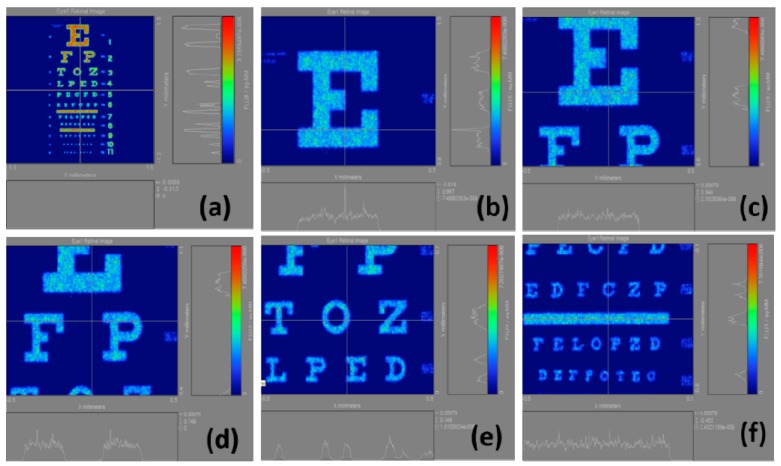
Imaging results of different regions selected in the Snellen chart calculated under standard normal vision conditions. (**a**) a whole location; (**b**) partial location: E; (**c**) partial location: E, F, and P; (**d**) partial location: E, F, P, T, O and Z; (**e**) partial location: F, P, T, O, Z, L, P, E and D; (**f**) partial location: lines 5–8 in the Snellen chart.

In this study, we calculate the total light flux in each image. In different myopia conditions, each image is expected to have different total light fluxes. We use the change in total light flux and the corresponding myopia to identify the relationship between them. Correspondingly, six regions of retinal image are obtained, as shown in Figure 3a–f. A whole or partial location in the Snellen chart is selected to calculate the retinal image. The result of the Snellen chart is illustrated on the left sides of the figures. In the left image, *x* is the spatial scale in direction *x*, and *y* is the spatial scale in direction *y*. In the right image, *x* is the light flux of the level, and *y* is the light flux corresponded by a perpendicular line. The right color bar is the energy distribution of the Snellen chart. Taking Figure 3a as an example, the right side refers to the light flux of the perpendicular line. When the perpendicular line passes through the image, the light flux significantly changes. In this case, the lower part represents the horizontal light flux. However, the horizontal line does not pass through any power in Figure 3a.

**Figure 4:**
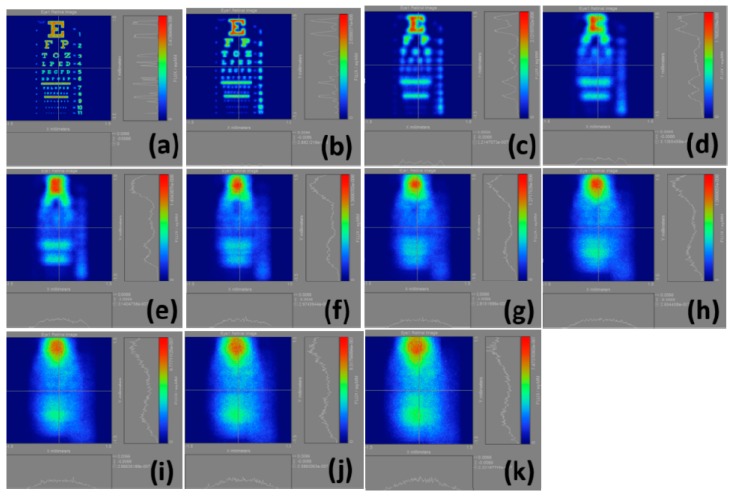
Calculation results of retinal images by selecting the whole Snellen chart in Figure 3(a); (**a**) plano; (**b**) 1.00 D; (**c**) 2.00 D; (**d**) 3.00 D; (**e**) 4.00 D; (**f**) 5.00 D; (**g**) 6.00 D; (**h**) 7.00 D; (**i**) 8.00 D; (**j**) 9.00 D; (**k**) 10.00 D.

The intensities of the color bars used in these figures also vary, in other words, different colors correspond to different intensities. If we regularize all intensities of the color bars, then the various powers of myopia would not be properly presented. Figure 4 illustrates the calculation results of the retinal images we made by selecting the whole Snellen chart in Figure 3a from the normal vision to a 10.00 D myopia. The results are shown in Figure 4a–k, indicating that the visual resolution begins to decline with the increase of myopia. Accordingly, the images gradually become blurred, and the corresponding total light flux changes.

**Figure 5:**
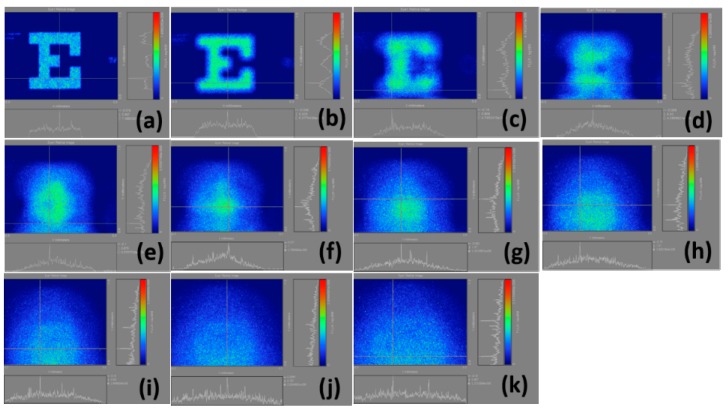
Calculation results of retinal images by selecting the whole Snellen chart in Figure 3(b); (**a**) plano; (**b**) 1.00 D; (**c**) 2.00 D; (**d**) 3.00 D; (**e**) 4.00 D; (**f**) 5.00 D; (**g**) 6.00 D; (**h**) 7.00 D; (**i**) 8.00 D; (**j**) 9.00 D; (**k**) 10.00 D.

The findings of this study are presented in several parts. Figure 5 shows the calculation results of the retinal images we made by selecting the regions in Figure 3b from the normal vision to a 10.00 D myopia. In particular, the results are displayed in Figure 5a–k which verify that as myopia increases, the visual resolution begins to deteriorate, and the images become blurred. However, the light intensity in Figure 5b is obviously visually higher than that in Figure 5a. The reason behind this condition is the fact that we do not normalize the intensity quantization resulting in the wrong visual judgment. The blurred images change the corresponding total light flux, and the decrease of light flux in the retina corresponds to the increase of the myopia power. 

**Figure 6:**
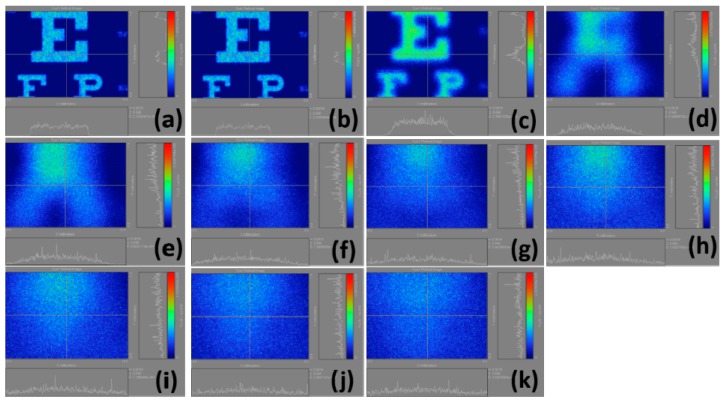
Calculation results of retinal images by selecting the whole Snellen chart in Figure 3(c); (**a**) plano; (**b**) 1.00 D; (**c**) 2.00 D; (**d**) 3.00 D; (**e**) 4.00 D; (**f**) 5.00 D; (**g**) 6.00 D; (**h**) 7.00 D; (**i**) 8.00 D; (**j**) 9.00 D; (**k**) 10.00 D.

**Figure 7:**
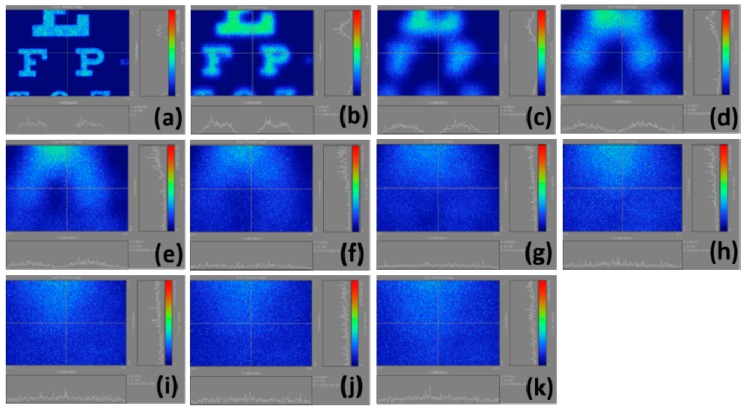
Calculation results of retinal images by selecting the whole Snellen chart in Figure 3(d); (**a**) plano; (**b**)1.00 D; (**c**) 2.00 D; (**d**) 3.00 D; (**e**) 4.00 D; (**f**) 5.00 D; (**g**) 6.00 D; (**h**) 7.00 D; (**i**) 8.00 D; (**j**) 9.00 D; (**k**) 10.00 D.

**Figure 8:**
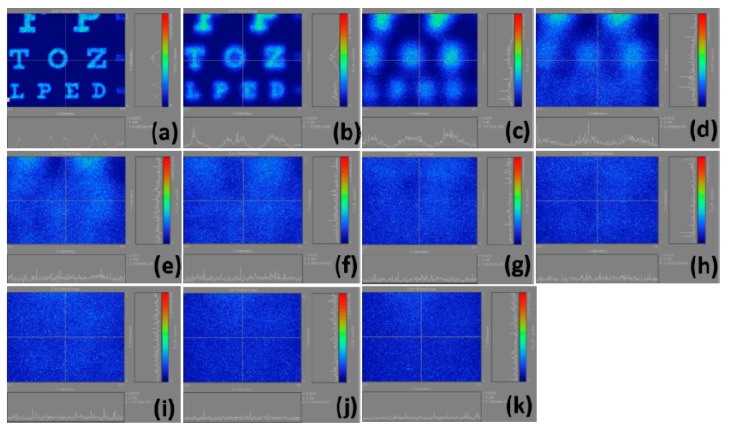
Calculation results of retinal images by selecting the whole Snellen chart in Figure 3(e); (**a**) plano; (**b**)1.00 D; (**c**) 2.00 D; (**d**) 3.00 D; (**e**) 4.00 D; (**f**) 5.00 D; (**g**) 6.00 D; (**h**) 7.00 D; (**i**) 8.00 D; (**j**) 9.00 D; (**k**) 10.00 D.

**Figure 9:**
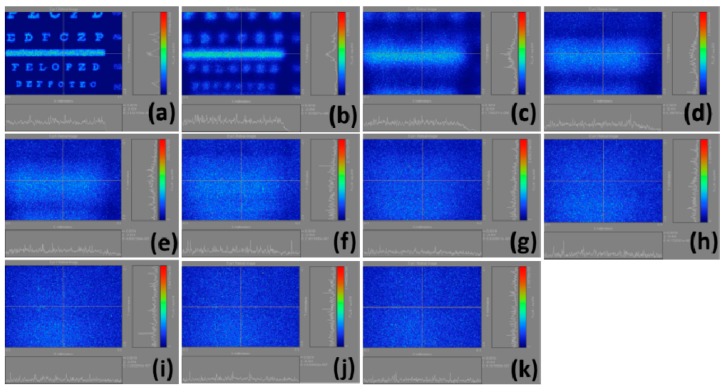
Calculation results of retinal images by selecting the whole Snellen chart in Figure 3(f); (**a**) plano; (**b**)1.00 D; (**c**) 2.00 D; (**d**) 3.00 D; (**e**) 4.00 D; (**f**) 5.00 D; (**g**) 6.00 D; (**h**) 7.00 D; (**i**) 8.00 D; (**j**) 9.00 D; (**k**) 10.00 D.

Figure 6 demonstrates the calculation results of the retinal images we made by selecting the regions in Figure 3c. In Figure 6b, the light intensities are strong, but they are rapidly decreasing; this is because our scales are not united and distinguishing these figures visually is difficult. Figure 7 shows the calculation results of the retinal images we made by selecting the regions in 3d. Similar to Figure 6b, the problem of Figure 7b is that the scale is not united, causing differences in light intensity visually. Figure 8 displays the calculation results of the retinal images we made by selecting the regions in Figure 3e. In Figure 8, the uncoordinated scale is not a significant problem. Moreover, this figure clarifies that the light intensity slowly weakens with the increase of myopia power. Figure 9 shows the calculation results of the retinal images we made by selecting the regions in Figure 3f. The results definitely show that the images become blurred with as myopia increases, and the corresponding light intensities are reduced. 

**Figure 10:**
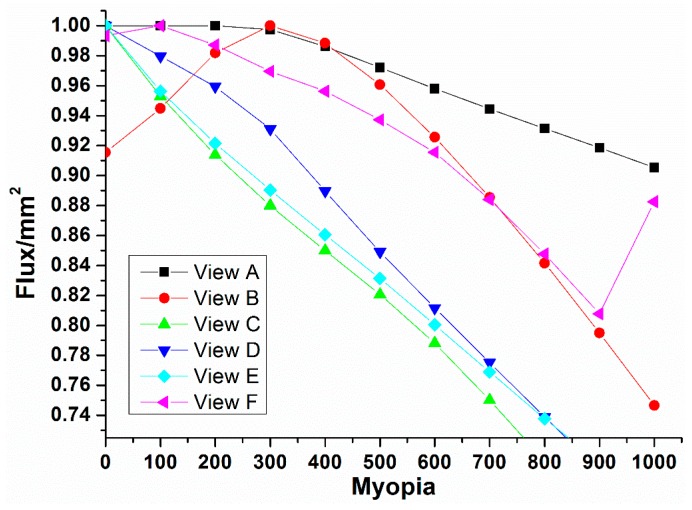
Effects of degrees of myopia on the changes in light flux in the visual region under different Snellen chart visual regions.

Meanwhile, Figure 10 shows the calculation results of light flux through all the results indicated in Figure 3a–f, which correspond to different refractive errors. When selecting Figure 3a, its result is shown as the curve of View A, which in turn, decreases with the increase of myopia. When selecting Figure 3b its result is shown as the curve of View B, which moves from low to high and then continuously declines with the myopia increase. This can be explained by the fact that with the change in myopia power, the stray light outside the selection region may affect the light flux in the selection region, making it unstable and causing sudden fluctuations. When selecting Figure 3c–e, their results are shown as the curves of Views C, D and E, respectively, with declining trends on the refractive error. When selecting Figure 3f, its result is the same as View F, which deteriorates with the increase of myopia, but suddenly rises at the final range of 9.00 D to 10.00 D. This can be attributed to the effect of the stray light outside the selection region on the light flux in the region.

Finally, we select View A as the standard for predicting cataracts. Table 1 shows the parameters for this calculation. In particular, Table 1 displays 11 different calculation results numbered from D1 to D11, respectively, two kinds of parameters (*i.e.*, 50 × 50 and 100 × 100 nanograins) in the Number column, and three levels. 

**Table 1:** Parameters for optical calculation.

Number	Array	Layer	D (mm)	Area (mm^2^)	Myopia
D1	100 × 100	1	0.02	4.0	—
D2	100 × 100	1	0.03	9.0	—
D3	100 × 100	1	0.04	16.0	—
D4	100 × 100	1	0.05	25.0	250
D5	50 × 50	2	0.07	24.5	450
D6	50 × 50	2	0.06	18.0	500
D7	50 × 50	2	0.05	12.5	580
D8	50 × 50	2	0.04	8.0	630
D9	50 × 50	2	0.03	4.5	680
D10	50 × 50	2	0.02	2.0	750
D11	100 × 100	2	0.04	32.0	850
D12	50 × 50	3	0.04	12.0	970

“Number” represents the calculations of different parameters; “array” represents the number of the adopted periodic hole arrays; “layer” refers to the number of the levels of periodic hole arrays made on the anterior surface of lens; *D* is the period of hole; and “area” refers to the distribution area of periodic hole arrays.

**Figure 11:**
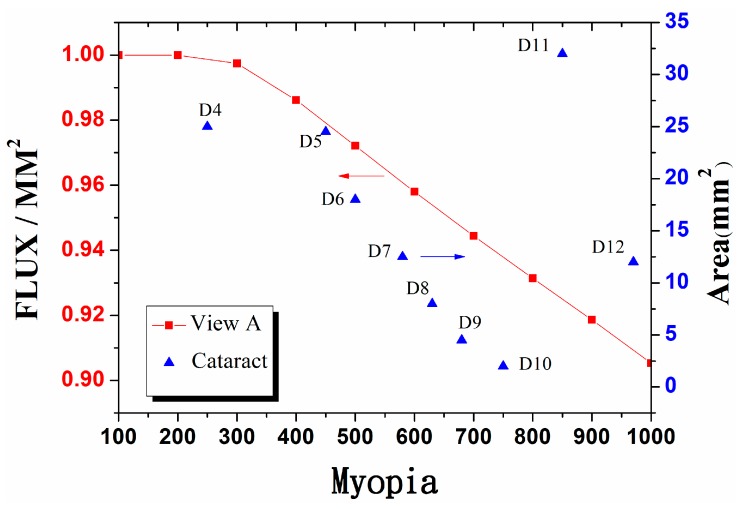
According to the calculation results of the parameters in Table 1, the blue spot refers to the area in Table 1. This is defined as the area of periodic structure multiplied by the number of levels.

The interval between the nanograins ranges from 0.02 mm to 0.07 mm. The surface area, which is equal to the number plus the interval multiplied by the length of side multiplied by the number of levels, is the area named. The degree is the light flux corresponding to different refractive error in Figure 3a to mutually correspond to the calculation results after adding the microstructure. The result is shown in Table 1, whose calculation results are presented in Figure 11. 

In this figure, the red curves depict the different levels of myopia power we set under the conditions of Figure 3a, thus obtaining different light fluxes. In this case, the light flux is normalized as 1. Thus, the total light flux in the retina is reduced with as the degree of myopia rises. The blue spot refers to the area in Table 1 and is defined as the area of periodic structure multiplied by the number of levels. If the area is large, then the myopia power is not high. By contrast, a small area is centralized in the visual center, leading to a high power of myopia. When some periodic structures are generated, they cause vision decline. The optical calculation model presented in this study can be used to predict the severity of lens lesions of patients suffering from a cataract. The amount of decreased light and the glare by light scattering will generate a condition similar to the myopia. Disability glare results from light scattering in the eyes. No international related method for measuring disability glare is currently available along with methods of measuring the severity of glare. One illustration is to take the correlation between the visual contrast and patient perception to glare if a participant with reduced visual contrast may exhibit increased disability glare. When the contrast is lower than the limits of human eye recognition, participants experience difficulty in distinguishing the objects. Therefore, disability glare must be accepted as it is an undeniable factor that can affect our vision in the future. 

## 4. Conclusions

The images in the retina are established by applying the ray tracing technique combined with a human eyeball module. Different regions in the Snellen chart are used to establish retinal images in different regions. The results revealed that the selection region may be affected by the stray light; hence, the whole Snellen chart is selected for simulation. In the microstructure, 50 × 50 and 100 × 100 nanograins exist. Under the 100 × 100 nanograin conditions, if the interval is set to be less than 0.05, then the corresponding degree cannot be distinguished. Contrarily, in the 50 × 50 two-level structure, the corresponding degrees to the intervals ranging from 0.02 to 0.07 can be identified. The myopia power can be used to predict the severity of lens lesions. If the distribution area of the microstructure is large, then the myopia power will not increase. However, a small area is centralized in the visual center, thereby increasing the myopia power.
